# “On-The-Fly” Calculation of the Vibrational Sum-Frequency Generation Spectrum at the Air-Water Interface

**DOI:** 10.3390/molecules25173939

**Published:** 2020-08-28

**Authors:** Deepak Ojha, Thomas D. Kühne

**Affiliations:** 1 Dynamics of Condensed Matter and Center for Sustainable Systems Design, Chair of Theoretical Chemistry, Department of Chemistry, Paderborn University, Warburger Str. 100, 33098 Paderborn, Germany; deepak.ojha@chem.uzh.ch; 2 Paderborn Center for Parallel Computing and Institute for Lightweight Design, Paderborn University, Warburger Str. 100, D-33098 Paderborn, Germany

**Keywords:** vSFG, air-water interface, on-the-fly, AIMD

## Abstract

In the present work, we provide an electronic structure based method for the “on-the-fly” determination of vibrational sum frequency generation (v-SFG) spectra. The predictive power of this scheme is demonstrated at the air-water interface. While the instantaneous fluctuations in dipole moment are obtained using the maximally localized Wannier functions, the fluctuations in polarizability are approximated to be proportional to the second moment of Wannier functions. The spectrum henceforth obtained captures the signatures of hydrogen bond stretching, bending, as well as low-frequency librational modes.

## 1. Introduction

Vibrational spectroscopy provides microscopic fingerprints of the structure and dynamics at the molecular level in condensed phase systems [1,2,3]. However, theoretical interpretation and peak characterization of vibrational spectra predominantly rely on molecular dynamics simulations [4,5,6,7,8,9]. Nevertheless, the success of simulations also depends largely on the force field employed to describe the interatomic interactions. In this regard, ab initio molecular dynamics (AIMD) has proven to be extremely useful as the interatomic forces are obtained from accurate electronic structure calculations [10,11]. For periodic systems, the overall electronic state within the AIMD framework is generally expressed in the terms of Bloch orbitals,

(1a) $$\Psi \left(r,k\right)=e^{\left(ik\cdot r\right)}\;u_{i}\left(r,k\right),$$

where

(1b) $$u_{i}\left(r,k\right)=u_{i}\left(r+R,k\right),$$

with Ψ(r,k) being the electronic wavefunction, $u_{i}\left(r,k\right)$ the Bloch function, and **R** a translational lattice parameter [12]. An alternative representation, which is more suited for chemical problems, is provided by so-called maximally localized Wannier functions (MLWFs), i.e., $w_{n}\left(r-R\right)$ that are obtained by a unitary transformation of the Bloch orbitals [13,14]. The construction of this Wannier representation enables to split the continuously varying total electronic density into contributions originating from localized fragments of the system. Mathematically, MLWFs are expressed as

(2) $$w_{n}\left(r-R\right)=\frac{V}{2\pi^{3}}\int_{BZ}dk\;e^{-ik\cdot R}\sum_{m=1}^{J}U_{mn}^{\left(k\right)}\psi_{mk}\left(r\right),$$

where R is the lattice vector of the unit cell and *V* is the real-space primitive cell volume. The J×J matrix $U_{mn}^{\left(k\right)}$ is the unitary transformation matrix and $\psi_{mk}\left(r\right)$ are the eigenstates of the system computed by density function theory (DFT). The corresponding MLWFs are then obtained by the unitary transformation $U_{mn}^{\left(k\right)}$ that minimizes the spread functional

(3) $$S=\sum_{n}S_{n}=\sum_{n}\left(\langle w_{n}\left|r^{2}\right|w_{n}\rangle -{\langle w_{n}\left|r\right|w_{n}\rangle }^{2}\right).$$

Therein, $\langle r^{2}\rangle$ is the second moment, whereas ${\langle r\rangle }^{2}$ is the squared first moment of the Wannier centers. This unitary transformation-based localization can be readily implemented on the position operator $\hat{r}$ within the Wannier representation to obtain localized orbitals for a given periodic system of arbitrary symmetry [15,16,17]. As a result, the scheme can be used to compute the electronic contributions to the polarization of a system. Moreover, it also allows to calculate instantaneous fluctuations in the molecular dipole moment and within the linear-response regime and obtain the linear as well as nonlinear infrared spectrum using time-correlation function formalism [18,19,20,21,22,23,24]. In this regard, Raman and higher nonlinear analogs like vibrational sum frequency generation (v-SFG), 2D-vSFG, and 2D-Raman can also be computed by applying a constant periodic electric field using the Berry phase formalism [25,26,27], or by calculating the polarizability tensor *A*

(4) $$A_{ij}=-\frac{\delta M_{i}\left(E\right)}{\delta E_{j}},$$

where *M* is the total dipole moment and *E* is an externally applied electric field. This scheme of computing the polarizability tensor has been utilized to obtain isotropic Raman spectrum by means of density functional perturbation theory [28,29,30,31].

In this paper, we present a novel computational method to obtain the v-SFG spectrum of the air–water interface. This anisotropic Wannier Polarizability (WP) method is based on a technique of computing the fluctuations within the dipole moment and polarizability “on-the-fly” during an AIMD simulation without any additional computational cost [32]. For that purpose, the fluctuations in the dipole moment are obtained using the Wannier centers, whereas the components of the polarizability tensor are approximated using the second moment of the Wannier centers. However, it is noteworthy that several other computational studies have obtained the vSFG spectrum using empirical maps [33,34,35,36,37,38,39,40], velocity correlations [41,42,43,44], as well as directly from AIMD simulations [45,46,47,48].

## 2. Results

### 2.1. Anisotropic Wannier Polarizability Method

The original isotropic WP method has been implemented to compute the isotropic Raman spectrum of isolated gas phase molecules, as well as aqueous solutions [32,49]. The underlying principle of the method is that the polarization induced by an externally applied perturbation is directly proportional to the molecular volume of the system [50,51]. As Wannier centers provide a picture where the total electronic density is partitioned into the localized electronic densities of different fragments of the system, the fluctuations in the electronic polarizability can be connected to the fluctuations of the volume of the Wannier centers instead of the overall molecular volume. As a result, the net isotropic polarizability can be expressed as

(5) $$\bar{A}=\frac{1}{3}\sum_{i=1}^{N_{WF}}A_{i}=\frac{\beta }{3}\sum_{i}^{N_{WF}}S_{i}^{3},$$

where $S_{i}$ is the spread of the *i*th Wannier center, $N_{WF}$ is the number of MLWFs, and β is a proportionality constant. The isotropic Raman spectrum is then obtained as the Fourier transform of the polarizability time-correlation function. On similar lines, the v-SFG spectrum of a non-centrosymmetric system is given by

(6) $$\chi_{abc}^{2}\left(\omega \right)=\int_{0}^{\infty }dte^{i\omega t}\langle M_{c}\left(0\right)\cdot A_{ab}\left(t\right)\rangle ,$$

which can be rewritten as

(7) $$\chi_{abc}^{2}\left(\omega \right)=\frac{1}{i\omega^{2}}\int_{0}^{\infty }dte^{i\omega t}\langle {\dot{M}}_{c}\left(0\right)\cdot {\dot{A}}_{ab}\left(t\right)\rangle ,$$

where $\chi_{abc}^{2}$ is the second-order susceptibility, whereas $A_{ab}$ is the abth component of the polarizability tensor and $M_{c}$ is *c*th component of the dipole moment [41]. In contrast to Raman spectroscopy, the computation of v-SFG spectra requires the diagonal elements of the polarizability tensor. In this regard, we note that the second moment $\langle w_{n}\left|r_{\mathrm{ab}}^{2}\right|w_{n}\rangle$ and the polarizability are tensors of same size. Moreover, the off-diagonal elements of the second moment should correspond to the time-dependent evolution of the off-diagonal polarizability tensor components. Accordingly, we have approximated that the component specific fluctuations in the polarizability are proportional to the second moment of the Wannier centers, i.e.,

(8) $$A_{ab}\propto \langle w_{n}\left|r_{\mathrm{ab}}^{2}\right|w_{n}\rangle .$$

This is to say that the correspondence between the second moment of the Wannier centers and the polarizability tensor allows us to approximate the fluctuations within the polarizability without actually calculating the exact numerical value of each component of the polarizability tensor. The strength of the anisotropic WP method is that for each set of an electron pair, we have a unique Wannier center and its corresponding moments. As a result, the method can be used to specifically study the contributions from the different fragments of the system. Moreover, it is also computationally less expensive as the polarizability is determined on-the-fly from the second moments of the Wannier centers, which is in contrast with existing approaches, where the polarizability is obtained by numerical differentiation of the total dipole moment with respect to an externally applied electric field. This is to say that a simple minimization of the spread functional provides the Wannier centers and their corresponding moments that are used to obtain the dipole and the polarizability, respectively. Thus, a single AIMD-based Wannier center calculation is sufficient to obtain the dipole moment, as well as the polarizability.

### 2.2. Application to the Air–Water Interface

**Figure 1:**
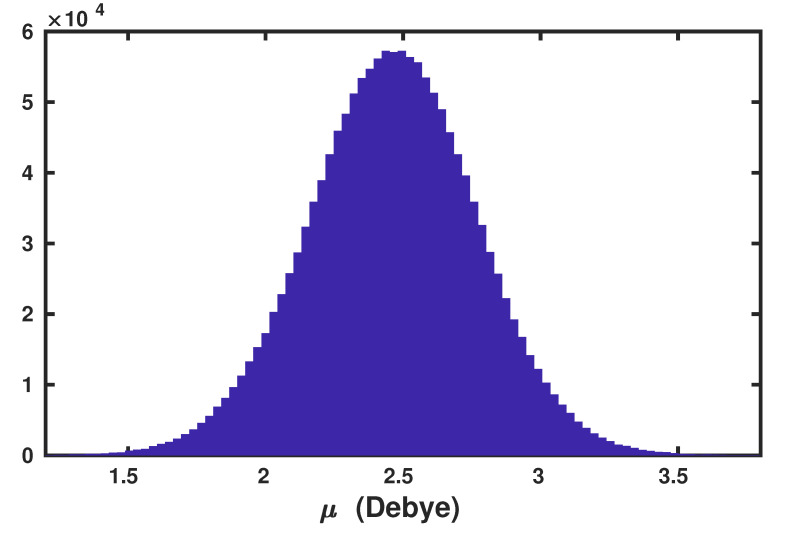
Distribution of molecular dipole moment (μ) of water molecules at ambient conditions, as computed using the maximally localized Wannier centers.

To demonstrate the predictive power of the present anisotropic WP method, we have computed the v-SFG of at the air–water interface. For the sake of simplicity, we have assumed that the contributions originating from Wannier centers, which are associated with the lone pair of electrons, to the overall polarizability can be safely neglected. Moreover, the spectral dynamics is predominantly governed by the dynamical evolution of the Wannier centers corresponding to the bonded electron pairs. This assumption is based on the fact that the time-dependent evolution of the Wannier centers with respect to centers of molecules corresponding to the bonded electron pairs is mutually complementary to that of the lone pairs. For timescales at around 1 ps, which is the timescale of hydrogen bond rearrangements, the strengthening of the donating hydrogen bonds leads to shift of the Wannier centers associated with bonded electron pairs away from the centers of water molecules, and vice versa for the Wannier centers corresponding to the lone pairs. The average molecular dipole moment of the water molecules obtained using the Wannier centers, whose distribution is shown in Figure 1, was found to be 2.46 Debye.

**Figure 2:**
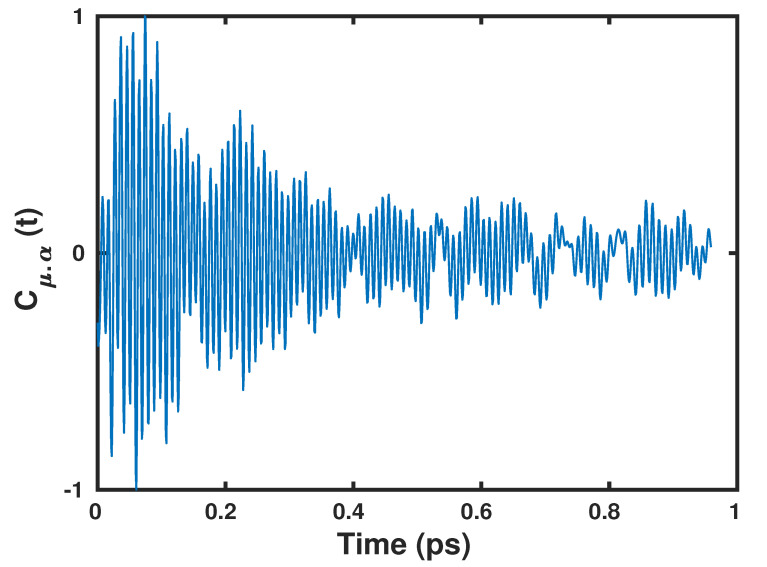
The dipole polarizability cross-correlation function, as obtained by the present anisotropic Wannier Polarizability (WP) method.

**Figure 3:**
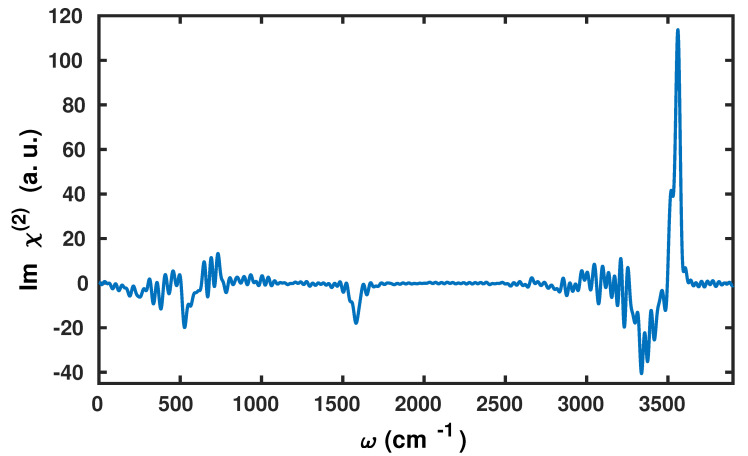
The vibrational sum frequency generation (v-SFG) spectrum of interfacial water molecules computed by the present anisotropic WP method.

The dipole polarizability cross-correlation function and the v-SFG spectrum computed based on the fluctuations within the dipole moments obtained by using the Wannier centers and polarizabilities by means of the second moment are shown in Figure 2 and Figure 3, respectively.

We find that the v-SFG spectrum obeys characteristic peaks corresponding to librational, bending, OH stretching, as well as free OH modes. As there are various previous experimental and simulation-based studies analyzing the stretching, bending, and librational modes within the v-SFG spectrum at the air–water interface, we only briefly highlight our findings in light of the existing literature. First, we will focus on the spectral region of 3000–3800 ${\mathrm{cm}}^{-1}$, which is predominantly attributed to OH stretching modes. More precisely, earlier simulation studies have reported a broad negative peak between 3000 to 3600 ${\mathrm{cm}}^{-1}$ and a sharp positive peak around 3700 ${\mathrm{cm}}^{-1}$ [33,34,36,37,45]. Using our anisotropic WP method, we also find a broad negative peak at 2900–3500 ${\mathrm{cm}}^{-1}$ and sharp positive peak around ∼$3600\;{\mathrm{cm}}^{-1}$. The former broad negative peak contribution originates from hydrogen-bonded water molecules with the overall dipole aligned towards the bulk, whereas the latter sharp positive peak is connected with the free and dangling OH modes of the interfacial water molecules.

The observed red-shift within the peak positions can be most likely attributed to the choice of XC functional and employed pseudopotentials in the present study. Earlier experimental and simulation studies of the bending mode have reported a broad negative peak around 1650 ${\mathrm{cm}}^{-1}$ and a positive shoulder around 1750 ${\mathrm{cm}}^{-1}$ [43,46]. Here, using the anisotropic WP method, we also observe a broad negative peak between 1400 and 1650 ${\mathrm{cm}}^{-1}$, which is governed by the free and dangling OH modes. However, at variance to these earlier studies [43,46], we cannot confirm any positive shoulder in our calculations. Finally, we observe a negative peak at around 450–650 ${\mathrm{cm}}^{-1}$ that is governed by the librational motion of water molecules.

**Figure 4:**
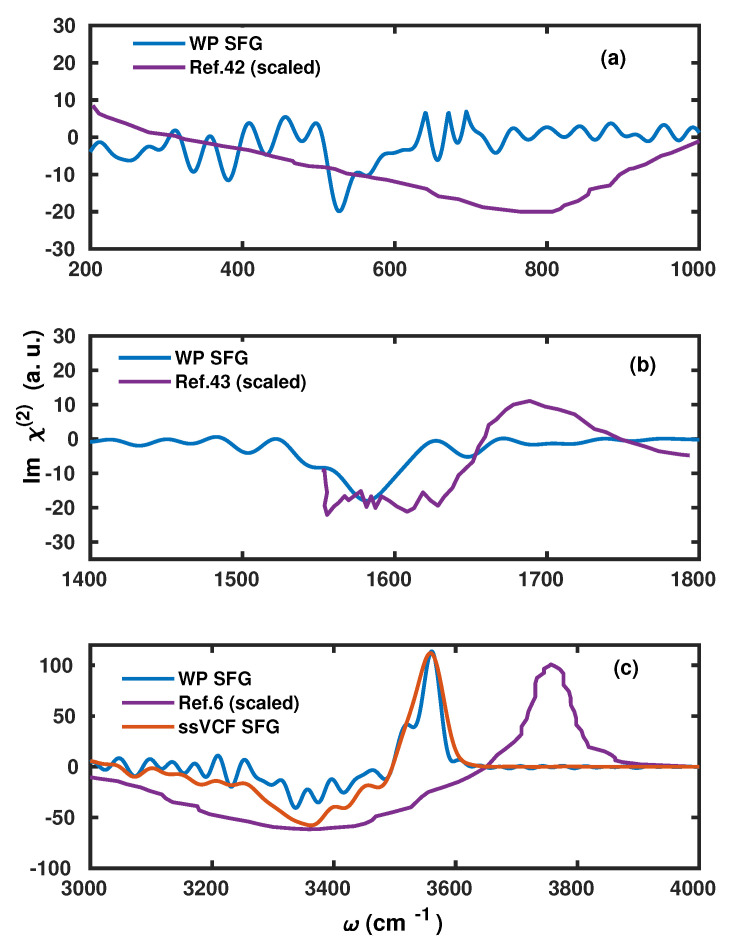
Comparison between the vSFG spectra as obtained using our WP method and experimental and theoretical measurements of (**a**) librational, (**b**) bending, and (**c**) stretching modes, respectively. The intensities of the experimental references were rescaled to match the theoretically obtained spectra.

However, we find it important to emphasize that the present scheme slightly underestimates the contributions originating from the bonded OH modes. This accounts for a shallow negative peak in the stretching region and a missing positive shoulder in the bending region. Apart from a consistent red-shift within the peak positions, our results are in good agreement with earlier results that have also reported a negative peak in the region of 700–800 ${\mathrm{cm}}^{-1}$ [42]. In Figure 4, we have compared the spectral line shape with available experimental results [6,42,43]. For the purpose to gauge the accuracy of the present method, we have also compared the stretching mode with the one obtained by the surface-specific velocity–velocity correlation function (ssVVCF) based on exactly the same trajectory [41,44].

## 3. Computational Methods

Ab initio molecular dynamics simulations were performed by using the method of Car and Parrinello [10,52], as implemented in the CPMD code [53]. Simulations of the air–water interface comprising of 80 $H_{2}O$ molecules were performed at 300 K in a cubic box of edge length 12.43 Å corresponding to the density at ambient conditions [54]. The air–water interface was generated by increasing the edge length of the box to 37.2 Å in the z-direction. The Kohn–Sham formulation of density functional theory was applied to represent the electronic structure of the system within a plane wave basis set [11]. In order to represent the core–shell electrons, Vanderbilt ultra-soft pseudopotentials were used and the plane wave expansion of Kohn–Sham orbitals was truncated at a kinetic energy cutoff of 25 Ry [55]. The electronic orbitals were assigned a fictitious mass of 400 a.u. and equations of motion were integrated with a time step of 4 a.u.

In the present work, we have used the dispersion corrected BYLP-D exchange and correlation (XC) functional [56,57,58], as previous AIMD studies have shown that inclusion of London dispersion interactions not only improves the structure, but also predicts the dynamics, spectroscopy and phase diagram of ab initio water and aqueous solutions in better agreement with experiment [48,59,60,61,62]. The initial configuration was generated using classical molecular dynamics simulations. Subsequently, the production run was carried out in the canonical NVT ensemble using Nose-Hoover thermostats [63,64] for 50 ps.

The identification of interfacial water molecules at the air-water system was conducted using the algorithm for the identification of truly interfacial molecules ITIM [65,66]. This scheme uses a probe sphere to detect the molecules at the surface. The radius of the probe sphere was set to 2 Å which has been proven to be a good value for water [66]. A cut-off-based cluster search was also performed using 3.5 Å as a cut-off, which corresponds to the first minimum of the O⋯O radial distribution function in liquid water.

## 4. Conclusions

To summarize, we have proposed a computationally efficient on-the-fly method to determine the v-SFG spectrum for interfacial systems. This anisotropic WP method utilizes the second moment of the Wannier centers to estimate the polarizability fluctuations. The major strength of this method is that it captures the spectral signatures of the system for the collective, as well as highly localized modes. Furthermore, it can be directly applied to spectral decomposition by computing fragment-specific contributions from the Wannier centers and their second moment to assist the interpretation of the experimental measurements. Moreover, the algorithm employed here can be easily extended to other spectroscopic techniques like two-dimensional v-SFG [67], time-dependent v-SFG [68], 2D-Raman-Thz [69], pump-probe Thz [70], and 2D-Raman [71] to name just a few. From the application perspective, interfacial reactivity, on-water catalysis, and other interfacial chemical processes can also be studied using our anisotropic WP-based method. Nevertheless, for greater agreement with the experiment, it would be important to better understand the role of simulation protocols, system size, and the approximations made, which we propose as an extensions for future works.
